# Hepatitis C Virus (HCV) Infection and Neurocognitive Impairment in Subjects with Mild Liver Disease

**DOI:** 10.3390/jcm12123910

**Published:** 2023-06-08

**Authors:** Marcia Maria Amendola-Pires, Max K. Fakoury, Hellen Salazar, Silvia B. De Oliveira, Carlos Eduardo Brandão-Mello, Sergio L. Schmidt

**Affiliations:** 1 Postgraduate Program in Neurology, Neurobehavioral Laboratory, Department of Neurology, Federal University of the State of Rio de Janeiro, Rio de Janeiro 20270-901, Brazil; mmamendola@gmail.com (M.M.A.-P.); max.fakoury@unirio.br (M.K.F.); hrmsalazar@hotmail.com (H.S.); psi.silviaoliveira@gmail.com (S.B.D.O.); cedubrandao@gmail.com (C.E.B.-M.); 2 Gastroenterology & Liver Unit, Gaffrée e Guinle University Hospital, Federal University of the State of Rio de Janeiro, Rio de Janeiro 20270-901, Brazil; 3 Internal Medicine Department, Gaffrée e Güinle University Hospital, Federal University of the State of Rio de Janeiro, Rio de Janeiro 20270-901, Brazil

**Keywords:** HCV (Hepatitis C Virus), cognitive disorder, verbal fluency, Symbol Digit Modalities Test, CVAT, cognitive impairment

## Abstract

Hepatitis C virus (HCV) infection is a leading cause of liver cirrhosis, hepatocellular carcinoma, and liver-related deaths. It is estimated that 40–74% of patients with hepatitis C will experience at least one extrahepatic manifestation within their lifetime. The finding of HCV-RNA sequences in post-mortem brain tissue raises the possibility that HCV infection may affect the central nervous system and be the source of subtle neuropsychological symptoms, even in non-cirrhotic. Our investigation aimed to evaluate whether asymptomatic, HCV-infected subjects showed cognitive dysfunctions. Twenty-eight untreated asymptomatic HCV subjects and 18 healthy controls were tested using three neuropsychological instruments in a random sequence: Symbol Digit Modalities Test (SDMT), Controlled Oral Word Association Test (COWAT), and Continuous Visual Attention Test (CVAT). We performed depression screening, liver fibrosis assessment, blood tests, genotyping, and HCV-RNA viral load. A MANCOVA and univariate ANCOVAS were performed to examine group differences (HCV vs. healthy controls) in four scores of the CVAT (omission errors, commission errors, reaction time—RT, and variability of RT—VRT), and the scores derived from the SDMT, and the COWAT. A discriminant analysis was performed to identify which test variables effectively discriminate HCV-infected subjects from healthy controls. There were no group differences in the scores of the COWAT, SDMT, and in two variables of the CVAT (omission and commission errors). In contrast, the performance of the HCV group was poorer than the controls in RT (*p* = 0.047) and VRT (*p* = 0.046). The discriminant analysis further indicated that the RT was the most reliable variable to discriminate the two groups with an accuracy of 71.7%. The higher RT exhibited by the HCV group may reflect deficits in the intrinsic-alertness attention subdomain. As the RT variable was found to be the best discriminator between HCV patients and controls, we suggest that intrinsic-alertness deficits in HCV patients may affect the stability of response times increasing VRT and leading to significant lapses in attention. In conclusion, HCV subjects with mild disease showed deficits in RT and intraindividual VRT as compared to healthy controls.

## 1. Introduction

Hepatitis C virus (HCV) infection is a blood-borne RNA virus of the genus *Hepacivirus* and family *Flaviviridae* with the potential to cause liver fibrosis, hepatocellular carcinoma, and liver-related deaths [1,2]. The global prevalence of viremic (HCV-RNA positive) HCV infection worldwide was estimated to be 0.7% (95% CI 0.7–0.9) at the beginning of 2020, corresponding to 56.8 million (95% CI 55.2–67.8) subjects [3,4]. In Brazil, the prevalence ranges from 0.7% to 1.2%, according to the Brazilian Ministry of Health [5]. Several studies indicated that approximately 60–85% of people exposed to HCV develop chronic infection. It is estimated that 40–74% of subjects infected with HCV will experience at least one extrahepatic manifestation within their lifetime [6,7,8,9,10,11].

Neurological complications are commonly seen in patients with liver disease. Chronic hepatic encephalopathy is associated with hepatic failure and portal-systemic shunting in cirrhosis and consists of an altered level of consciousness, asterixis, ataxia, confusion, spatial disorientation, and visual hallucinations [12]. In addition, selective impairments of psychomotor speed, visual perception, and attention have been reported in patients with cirrhosis without clinical signs of hepatic encephalopathy [13,14]. These selective impairments have been termed minimal hepatic encephalopathy (MHE) [13,14,15].

The finding of HCV genetic sequences in post-mortem brain tissue raised the possibility that HCV infection may affect the central nervous system and be the source of subtle neuropsychological symptoms in non-cirrhotic HCV patients [16,17,18,19]. Accordingly, it has been hypothesized that chronic HCV infection may result in cognitive impairment before the development of cirrhosis (20). To our knowledge, however, methodological limitations preclude a clear interpretation of putative cognitive impairment in HCV patients without cirrhosis.

Forton et al. [20] reported subtle neurocognitive impairment in a proportion of patients with histologically mild chronic HCV infection. A similar prevalence of cognitive dysfunction was reported by Hilsabeck et al. [21,22,23], but it was not clear whether the impairment was directly attributable to the HCV infection. Weissenborn et al. [24,25,26] described selective cognitive deficits associated with fatigue in patients with HCV infection without cirrhosis. In contrast, Cordoba et al. [27] did not find any evidence of cognitive impairment in HCV-infected patients without cirrhosis and stressed that impairments were only detected in patients with previous hepatic decompensation, which was caused by MHE. Abrantes et al. [28] using traditional screening instruments, such as the mini-mental status examination (MMSE), reported that there was not any evidence of an association between mild HCV infection and cognitive impairment. A narrative review by Abrantes et al. [29] concluded that there was not sufficient evidence in the literature to support that mild HCV infection could be associated with cognitive changes. Later, Abrantes et al. [30] described a marginally significant difference in the MMSE scores between healthy controls and mild HCV asymptomatic patients. However, this finding should be interpreted with caution because the reported small difference in the MMSE was not clinically meaningful and did not reach significance after adequate corrections for multiple comparisons. Recently, specific cognitive impairments have been described in asymptomatic HCV patients, but the lack of laboratory results confirming the absence of human immunodeficiency virus (HIV) limits the interpretation of this finding [31].

Based on the above-mentioned literature, whether HCV infection could be a cause of cognitive alterations in asymptomatic subjects with mild liver disease is still a matter of controversy. This controversy may be explained by at least three issues. First, there are marked differences in sample selection among the different studies. Secondly, the criteria used to define cognitive impairment varied from one study to another. Thirdly, the neuropsychological batteries employed in some studies were not adequately validated to investigate hypothetical cognitive deficits in patients with chronic hepatitis C.

It is intuitive that the neuropsychological battery should include tests that involve the brain regions affected by HCV. Neurotropism induced by HCV infection is found in the frontal cortices, cingulate gyri, medulla, and basal ganglia [11,32,33]. In this regard, functional neuroimaging in Go/No-Go tests has demonstrated the activation of the cingulate cortex and frontoparietal attention networks [34,35].

The Continuous Visual Attention Test (CVAT) is a Go/No-Go test commonly used in neurological patients [36,37,38,39,40]. Previous studies in patients and healthy subjects have suggested that the CVAT can be used to identify attention subdomain impairments independent of participants’ schooling [37]. Therefore, the CVAT may be a clinically useful instrument to be administered in asymptomatic HCV patients. The possible cognitive dysfunction in non-cirrhotic patients without previous decompensation is expected to be mild and specifically related to the brain regions affected by the HCV virus.

Our investigation aimed to evaluate whether untreated asymptomatic HCV-infected subjects showed cognitive dysfunctions. We administered traditional neuropsychological instruments and the CVAT. Furthermore, we used stringent criteria for cognitive impairment and for the selection of HCV patients. We hypothesized that the patients will present clinically significant deficits in specific attention subdomains.

## 2. Materials and Methods

### 2.1. Study Design

We conducted this study to analyze and evaluate cognitive performance in 2 groups: clinically stable HCV carriers and healthy subjects without HCV (controls). Therefore, the study design could be characterized as a cross-sectional observational study in which the groups were identified and compared based on the presence of HCV.

### 2.2. Settings and Participants (Patients and Controls)

This study was conducted at the Liver Unit of Gaffrée e Guinle University Hospital (HUGG), a tertiary Hospital in Rio de Janeiro, Brazil, from May 2019 through March 2020. All HCV-infected subjects over 18 years old (anti-HCV reactive and with detectable HCV-RNA for more than six months) who were being tracked in the Liver Unit were considered eligible for investigation. Only HCV subjects without clinically significant manifestations were included in the patient group.

The control group consisted of caregivers who voluntarily agreed to participate in the study. They were paired with the HCV group by age and Human Development Index (HDI) and only included subjects who had not had a previous infection with HCV and were over 18 years old.

The following exclusion criteria were established for both groups: less than 4 years of formal education; history of the encephalic vascular accident, encephalic cranial trauma, and loss of consciousness; systemic lupus erythematosus; Parkinson’s disease; neurological or neuropsychiatric disease (epilepsy, schizophrenia, depression, dementia, or multiple sclerosis); chronic obstructive pulmonary disease; congestive cardiac insufficiency; other viral infections (HIV, hepatitis B virus-HBV, or human T lymphotropic virus-HTLV); syphilis; advanced chronic liver disease with abnormal liver enzymes; cirrhosis; reduced kidney function; thyroid diseases; anemia with hemoglobin inferior to 9.5 g/dL; arterial hypertension not controlled by medication; non-corrected hearing or visual impairments; use of illicit and antipsychotic or anti-epileptic medication at any time or other psychotropic drugs that could affect attention performance; alcohol abuse; and any previous report of cognitive impairment.

HCV subjects were excluded if they showed clinically significant manifestations on the testing day. Patients with a history of previous hepatic decompensations were also excluded. Only HCV patients without any previous treatment were included in the final analysis. After the laboratory results more stringent exclusion criteria were applied, as outlined in the results section.

A sample of 48 HCV patients was initially evaluated. After applying the exclusion criteria, 20 patients were eliminated due to the following reasons: (a) previous use of α-interferon (n = 8); (b) cirrhosis (n = 3); (c) type 2 mellitus diabetes (n = 3); (d) depression (n = 2); (e) patients without assessment of renal function (n = 2); (f) hypothyroidism (n = 1); and (g) bridging fibrosis (F3 fibrosis) subject with three abnormal liver enzymes (n = 1). Then, twenty-eight clinically stable HCV-infected subjects and 18 healthy controls were and included in a detailed statistical analysis (item 2.4.4).

### 2.3. Procedures

All participants (48 HCV patients and 18 controls) were tested at the same moment using three neuropsychological instruments (Symbol Digit Modalities Test—SDMT, Controlled Oral Word Association Test—COWAT, and CVAT) in a random sequence. Depression screening, liver fibrosis assessment, and blood tests were performed. The subjects answered questions regarding predefined cognitive complaints (poor memory, dispersal/distractibility, and difficulty in performing two tasks simultaneously.

Patients and controls were paired by age, and geopolitical residence based on the HDI [41,42]. We also collected years of education in all participants for the purpose of comparison with the Brazilian norms.

#### 2.3.1. Depression Screening

Individuals with unipolar major depression episodes on the testing day were excluded. The presence of a mood depression episode was investigated with the aid of a structured clinical interview based on the DSM-V [43].

#### 2.3.2. Liver Fibrosis Assessment

HCV patients were previously evaluated to assess the grade of fibrosis by non-invasive tests (transient hepatic elastography-FIBROSCAN^®^ (Echosens, Paris, France)) or the AST to Platelet Ratio Index-APRI).

According to the Metavir score for grading fibrosis, subjects were categorized in the following scores: F0 (absence of fibrosis), F1 (minimal fibrosis), F2 (septa fibrosis), F3 (numerous septa portal-portal or portal vein fibrosis without cirrhosis) and F4 (cirrhosis) [44].

The APRI score represents the relationship between the platelet count and AST level. Levels ≥1.5 indicate the presence of significant fibrosis, whereas levels ≤0.5 points to the absence of significant fibrosis. Using a cutoff level of ≤1.0, we can rule out the presence of cirrhosis with a sensitivity of 89% and specificity of 75% [45].

#### 2.3.3. Blood Tests

Hematological and biochemical tests were performed at baseline, on all the HCV subjects to evaluate blood and platelet counts, liver inflammation (AST, ALT, and γGT), and function (prothrombin time, albumin, and bilirubin), as well as HCV genotyping, done by sequencing, to discriminate genotypes 1 or non 1 (genotypes 2–6).

Quantitative HCV-RNA viral load was locally performed by RT-PCR quantitative test (lower limit of detection: 12 IU/mL) (Cobas Monitor Roche) at baseline.

#### 2.3.4. Neuropsychological Instruments

The cognitive battery was performed in a quiet room by a medical doctor and psychologists with training in the application of neuropsychological tests and who were aware of the serological state of the individuals being tested. All the tests that were applied were translated into Portuguese.

The COWAT [46] is a battery with two tasks designed to assess verbal functioning. On each trial, the subjects were asked to generate as many Portuguese words as possible within 60 s. It consisted of two tasks: category fluency and letter fluency or phonemic fluency. In the category trials, the subjects were given 1 min to produce as many unique words as possible within a semantic category (animals) and then within another semantic category (fruits). Letter fluency was tested in the next three trials. First, the subjects were given 1 min to produce words starting with the letter F, then with the letter A, and finally with the letter S. For the three letter fluency subtests, the subjects were instructed that proper nouns were not allowed (places and people). For the category test, there was no restriction. For both tests (category and fluency), repetitions were not considered valid responses.

The SDMT is also a brief test [47]. The participants had 90 s to pair specific numbers with given geometric figures. Here, the subjects gave written responses. Each participant’s score was obtained by summing the number of correct substitutions within a 90-s interval.

**Figure 1:**
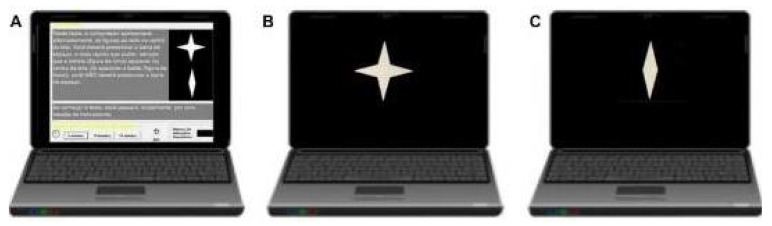
Schematic overview of the CVAT (Continuous Visual Attention Test, Go/No-go task of 15 min). Schematic overview of the CVAT showing the target (star) and non-target (diamond). The CVAT begins with written instructions on the screen (**A**) and a practice session. Subjects were instructed to use their dominant hands to press the spacebar whenever the star (**B**) appears in the center of the screen and should not press the spacebar when the diamond (**C**) appears. There were six blocks with three sub-blocks each of 20 trials (two figures presented whether targets or not). Three blocks had a 20% target probability, whereas, in the other three, the target probability was 80%. For each block, the sub-blocks had different interstimulus time intervals (ISI): 1, 2, or 4 s. The order of the ISI varied between blocks. Each stimulus was displayed for 250 milliseconds (ms). The total test took 15 min to complete. The types of measures included Omission Errors, Commission Errors, average Reaction Time, and Variability of Reaction Time.

The CVAT (Figure 1) consists of a Go/No-go task of 15 min composed of two figures, both colored white, which were presented sequentially in the center of a black screen. The testing equipment consisted of a laptop computer linked to a 13-inch performance liquid-crystal display. Subjects were seated in front of the computer in a way as to allow the hands to be comfortably placed over the keyboard. The distance from the eyes to the center of the monitor was approximately 50 cm. Before each task, the examiner instructed the subject to press the spacebar on the keyboard as fast as possible each time a specific visual target stimulus was displayed on the monitor. The test started with instructions and a practice session. They were instructed to use their dominant hands while performing the task. There were six blocks with three sub-blocks each of 20 trials (two figures presented whether targets or not). Each block had 60 stimuli: odd sequences composed of 16 target stimuli and 4 non-target stimuli (80% of target probability), and even sequences composed of 4 target stimuli and 16 nontarget stimuli (20% of target probability). For each block, the sub-blocks had different interstimulus time intervals (ISI): 1, 2, or 4 s. The order of the ISI varied between blocks. Each stimulus was displayed for 250 milliseconds. The total test took 15 min to complete. The types of measures included Omission Errors, Commission Errors, average Reaction Time (RT), and Variability of RT (VRT). VRT was estimated by a per-person measure of the standard deviation (SD) of individual RTs for the correctly signaled targets. To exclude the possibility that a participant’s VRT might be related to RT, we calculated the coefficient of variability (CV = VRT/RT) [37,48].

#### 2.3.5. Variables of the Study

This study analyzed the effect of the HCV (independent variable) on the variables corresponding to the neuropsychological tests (dependent variables). We applied age, sex, and HDI as confounders. We also analyzed the differences between controls and untreated asymptomatic HCV carriers without considering these three confounders.

Variables corresponding to neuropsychological tests (dependent variables)**:** COWAT performance was assessed by the verbal fluency variable which was calculated by the sum of the total number of words of the category tasks and the total number of words of the letter fluency task. The total number of correct answers was the variable used for measuring SDMT performance. The four sub-scores of the CVAT (omission errors, commission errors, RT, and VRT) were the variables used to measure four distinct attention subdomains.

Therefore, 6 variables with clear psychological meaning were used to evaluate whether there was any occult cognitive impairment in untreated asymptomatic HCV subjects.

### 2.4. Statistical Analysis

#### 2.4.1. Definition of Cognitive Impairment

This study used stringent criteria for cognitive impairment, by performing statistical analyses on raw scores compared with the healthy control group together with a careful analysis of the clinical relevance of the differences.

Some investigators defined objective cognitive impairment as a score that was 1.5 standard deviations (SD) or more below the normative mean on two or more tests measuring different cognitive domains [49,50,51]. Here, to better cover the range of possible diagnostic approaches, we considered that mild cognitive impairment with clinical significance required impairment only on one neuropsychological test variable, with impairment identified at the level of 1 SD below normative data [52].

#### 2.4.2. Sample Size Calculation (Power Analysis)

Following the STROBE statement of items that must be included in reports of observational studies, we included this separate section describing the way we conducted the sample size calculation [53]. To estimate the required sample size, we performed a power analysis considering α = 0.05 (Type I error) and β = 0.20 (Type II error). Thus power (1–β) was equal to 0.80.

In the present study, the main objective was to verify whether there was a difference in cognitive performance between untreated asymptomatic HCV carriers and healthy controls. As described below (Section 2.4.4), the comparison between the two groups was initially performed with the aid of a MANCOVA. However, irrespective of the results of the MANCOVA, we always performed post hoc tests to determine where there were significant differences in cognitive performance between untreated asymptomatic HCV subjects and controls. As all the post hoc comparisons were variations of t-tests, we performed power analyses considering independent t-tests.

We estimated the minimum differences (Δ) considering that they must reach magnitude levels that have clinical significance. According to our definition of mild cognitive impairment (Section 2.4.1), this difference should be at the minimum level of 1 SD below normative data. Therefore, for each variable of the neuropsychological tests, the population standard deviation (σ) and the mean difference (Δ) with a real clinical significance were estimated based on the normative values of the tests.

For the CVAT variables, we also evaluated clinically relevant differences based on larger samples in previous studies that evaluated the comparison between healthy controls and patients with Mild Cognitive Impairment as defined by the Clinical Dementia Rating Scale (CDR^®^). Therefore, for each variable of the CVAT, we also took into account a separate analysis considering the data derived from these studies (estimation of σ and Δ for each variable of the CVAT). We found the following values for the differences: ΔOE = 2 errors; ΔCE = 4 errors; ΔRT = 30 ms, and ΔVRT = 15 ms.

Then, we calculated all possible values for Cohen’s d [54]. Since the expected difference could be small or none, we selected the smallest clinically relevant effect size. Accordingly, we performed power analysis with the lowest Cohen’s d among all the variables, i.e., 0.98. For an allocation ratio of 2, we found the following sample sizes: n_group1_ = 13 and n_group2_ = 27.

#### 2.4.3. Descriptive Analysis: Demographics, Laboratory Findings, and Clinical Data

Participants included in this analysis: controls (n = 18), untreated asymptomatic HCV carriers (n = 28), and eliminated HCV patients (n = 20).

As described in Section 2.2, our study included a control group with 18 healthy subjects. From an initial sample of 48 HCV patients, we eliminated 20 patients after applying the exclusion criteria. Then, the descriptive analyses included these three groups: controls (n = 18), asymptomatic untreated HCV carriers (n = 28), and patients with HCV who were eliminated due to the exclusion criteria (n = 20). We performed comparisons in the demographic variables among these 3 groups. For the two HCV groups, we compared disease evolution time and viral genotype.

For the statistical comparisons outlined in the above paragraph, we used chi-squared tests for the dichotomic variables and t-tests for all continuous variables (K-S tests were performed to check normality assumptions).

Although we did not perform statistical comparisons in cognitive performance between the two HCV groups, we included all means and standard deviations of the unadjusted estimates of the 6 neuropsychological variables (raw scores). This allowed direct visualization of the cognitive performance of the two HCV groups and the controls.

The laboratory values of patients in the two HCV groups were compared with the normal range. This allowed the identification of participants outside the normal range.

#### 2.4.4. Main Objective: Quantitative Analysis of the Differences in Cognitive Performance between Untreated and Asymptomatic HCV Carriers (n = 28) and Healthy Controls (n = 18)

All the following statistical procedures were performed using the 4 variables directly obtained from the CVAT (Omission Errors, Commission Errors, RT, and VRT) and the two variables derived from the traditional tests (SDMT—number of correct answers, COWAT- total verbal fluency). Therefore, we used 6 dependent variables to evaluate cognitive performance.

A MANCOVA was performed to examine group differences (HCV vs. Healthy) in the six variables, using age, HDI, and sex as confounders. Box’s M test was used to assess the homogeneity of the covariance matrices. The Box’s M test was interpreted in conjunction with the inspection of the log determinants. Then, univariate ANCOVAs were performed to assess the effect of HCV (yes or no) on each one of the six neuropsychological variables. For the MANCOVA and for each one of the univariate ANCOVAs, η^2^ (Eta squared) was computed to calculate the effect size of the results. Cohen [54] suggested that η^2^ = 0.01 be considered a ‘small’ effect size, 0.06 represents a ‘medium’ effect size, and 0.14 a ‘large’ effect size.

In the present study, we balanced the groups considering potential confounders. Covariates and confounders are typically included in statistical models to account for the variance they explain in the dependent variables. This has a negative impact on the statistical power. However, matching can break the association between confounders and the variables of interest. Thus, as we have a matching design it was possible to perform the MANCOVA and respective ANCOVAs without confounders and covariates. Considering that this approach could increase the statistical power, we also performed the MANCOVA and respective ANCOVAs without confounders.

However, matching is more complex than just balancing groups for potential confounders. Some investigators [55] have proposed that the benefit obtained from accounting for the variance the confounders explain in the dependent variables is greater than the increase of power obtained without confounders. Therefore, a matched design may require controlling for the matching factors in the analysis. For this reason, we decided to present results with and without confounders.

Comparing averages can indicate variable between-group cognitive differences. However, it does not indicate which variables effectively discriminate between the two groups. Thus, a discriminant analysis was performed using the raw scores of the neuropsychological tests. Initially, the equality of the group means was tested using Wilk’s λ. A linear discriminant analysis was applied to find a linear combination of the variables that characterize or separate the two groups. The canonical discriminant function coefficients were calculated to obtain the discriminant function. Chi-squared tests (χ^2^) were performed to verify if the discriminant functions did better than the chance of separating the groups. With the aid of the function obtained, the accuracy of the classification was measured. Pearson correlations between predictors and standardized canonical discriminate functions were calculated.

For all the tests, significance was set at *p*-value <0.05 (bilateral). Corrections for multiple comparisons were performed with the Bonferroni method.

## 3. Results

### 3.1. Demographic and Clinical Data (Table 1, Table 2 and Table 3)

Demographic variables are described in Table 1. There were more females than males in the control group. Age, Years of education, and HDI did not differ among all groups (*p* > 0.05 for all comparisons).

**Table 1:** Demographic characteristics of HCV and control groups.

Demographic Characteristics	HCV Whole Sample (n = 48)	HCV Eliminated Group (n = 20)	HCV Mild Hepatitis Group (n = 28)	Control Group (n = 18)
Age (mean ± SD)	53.89 ± 12.64	57.85 ± 13.17	51.07 ± 11.69	42.83 ± 15.86
Female (n/%)	26 (54.16%)	9 (45%)	17 (60.71%)	17 (94.44%)
Years of Formal Education (mean ± SD)	11.77 ± 5.03	10.75 ± 5.32	12.5 ± 4.78	15 ± 3.37
HDI (mean ± SD)	0.81± 0.07	0.80 ± 0.07	0.81 ± 0.07	0.78 ± 0.05

HCV, Hepatitis C Virus; HDI, Human Development Index; SD, Standard Deviation. Continuous variables (age and HDI) are represented by mean ± SD. The hachured area indicates the data that will be used in the quantitative analysis comparing controls to mild HCV participants (reported in Section 3.2).

Table 2 shows the laboratory, virological, and epidemiological characteristics of the HCV patients. Genotype 1 was found in 82.14% of patients (23/28). Four patients had genotype 3, and one patient had genotype 4. Concerning the stage of fibrosis, 82.14% of the study group was composed of subjects with minimal or moderate fibrosis (F0–F2) and 5 (17.85%) with bridging fibrosis (F3). The mean HCV-RNA viral load was 2,165,514 IU/mL with a range of 9,805—19,277,067 IU/mL, being 18 out of 28 loads ranging from >500,000 and <6,000,000 IU/mL. The main form of HCV transmission was by blood transfusion (15 cases—53.57%) followed by sporadic/community (5 cases—17.85%). Due to the small number of subjects in some cells, statistical comparisons were not performed for a mode of transmission and grade of fibrosis. All other possible comparisons between HCV-mild hepatitis (n = 28) and HCV-eliminated (n = 20) did not reach significance (*p* > 0.5).

Table 3 indicates the values of the neuropsychological variables of all groups. Based on qualitative descriptive analyses we found that performance in all variables except verbal fluency (COWAT) was worse in the eliminated group (n = 20, which included some cirrhotic patients) as compared to the Mild HCV group. The same results were found when mild HCV participants were compared to controls. The poorest performance was always found in the eliminated group.

**Table 2:** Laboratory, virological, and epidemiological characteristics of the HCV samples.

Characteristics		HCV Whole Sample (n = 48)	HCV Eliminated Group (n = 20)	HCV-Mild Hepatitis Group (n = 28)
Disease Evolution Time	Years (mean ± SD)	12.08 ± 7.64	13.05 ± 7.78	11.39 ± 7.60
Transmission	Transfusion	25 (52.08%)	10 (50%)	15 (53.57%)
	Sporadic	12 (25%)	7 (35%)	5 (17.85%)
	Others	2 (4.16%)	-	2 (7.14%)
	Sexual	3 (6.25%)	1 (5%)	2 (7.14%)
	Illicit drugs	6 (12.5%)	2 (10%)	4 (14.28%)
Viral genotype	Genotype (1:non 1)	41:7	18:2	23:5
Grade of Fibrosis	Fibrosis (F0–F2)	36 (75%)	13 (65%)	23 (82.14%) 5 (17.85%)
	Fibrosis (F3)	6 (12.5%)	1 (5%)	
	Fibrosis (F4)	6 (12.5%)	6 (30%)	
Laboratory Value (mean ± SEM)	AST	50.21 ± 5.25	42.13 ± 5.55	55.73 ± 8.03
	ALT	61 ± 7.56	49.90 ± 6.96	67.77 ± 11.84
	GGT	73.28 ± 12.07	75.66 ± 16.92	72.04 ± 16.89
	TB	0.66 ± 0.04	0.66 ± 0.06	0.67 ± 0.06
	Albumin	4.23 ± 0.15	4.17 ± 0.12	4.26 ± 0.24
	Platelets	214,068 ± 10,361.22	188,333 ± 18,440.04	231,885 ± 11,070.85
	INR	1.03 ± 0.02	1.01 ± 0.01	1.04 ± 0.04
	Leukocytes	5470 ± 237.97	5427 ± 390.62	5499 ± 304.61
	Hemoglobin	13.91 ± 0.38	14.07 ± 0.43	13.80 ± 0.57
	Glucose	99.27 ± 5.40	116.35 ± 12.30	88.51 ± 2.29
	Urea	27.35 ± 1.29	31.07 ± 1.76	25.14 ± 1.65
	Creatinine	0.81 ± 0.02	0.84 ± 0.03	0.80 ± 0.03

HCV, Hepatitis C Virus; AST, Aspartate Aminotransferase (reference range <38 IU/mL); ALT, Alanine Aminotransferase; GGT, Gamma Glutamyl Transferase; TB, Total Bilirubin; SD, Standard Deviation; SEM, Standard Error of the Mean. Normal reference ranges: AST (<38 IU/mL); ALT (<41 IU/mL); GGT (<50 IU/mL); TB (≤1.3 mg/dL); Albumin (3.5–4.8 g/dL); Platelets (150,000–450,000 mm^3^); INR, International Normalized Ratio (0.85–1.30); Leukocytes (4500–10,000); Hemoglobin (12–16 g/dL); Glucose (70–99 mg/dL); Urea (10–50 mg/dL); Creatinine (0.6–1.1 mg/dL). All possible comparisons did not reach significance (*p* > 0.05).

**Table 3:** Raw Scores (unadjusted estimates) of CVAT, SDMT, Fluency Tests (Animal and Letters) HCV subjects versus Healthy controls.

Neuropsychological Battery (Mean ± SEM)		HCV—Initial Sample (n = 48)	HCV Eliminated Group (n = 20)	Mild Hepatitis HCV Group (n = 28)	Control Group (n = 18)
COWAT	FL (Animal + Fruit) + FAS	66.2 ± 2.80	64.5 ± 4.48	67.5 ± 3.63	67.9 ± 3.08
SDMT	Number of correct answers	32.35 ± 1.82	27.65 ± 2.85	35.71 ± 2.18	39.72 ± 2.56
	OE	2.87± 0.91	4.75 ± 1.88	1.54 ± 0.76	0.67 ± 0.30
CVAT	CE	6.14 ± 0.89	5.00 ± 0.95	6.96 ± 1.36	5.50 ± 0.59
	RT (ms)	453.83 ± 9.67	463.35 ± 16.90	448.18 ± 11.14	408.33 ± 6.84
	VRT (ms)	93.0 ± 5.54	99.05 ± 12.47	88.71 ± 3.46	78.94 ± 3.39

HCV, Hepatitis C Virus; CVAT, Continuous Visual Attention Test; ms, milliseconds; OE, Omission Error. CE, Commission Error; RT, Reaction Time; VRT, Variability of Reaction Time; COWAT, Controlled Oral Word. Association Test; FL, Fluency; SDMT, Symbol Digit Modalities Test; SEM, Standard Error of the Mean. The hachured area indicates the data that will be used in the quantitative analysis comparing controls to mild HCV participants (reported in Section 3.2).

### 3.2. Quantitative Analyses (Controls vs. Mild HCV)

#### 3.2.1. Average Differences in Neuropsychological Performance between Mild HCV and Controls (Figure 2)

As indicated in the methods section, we performed MANCOVAs and ANCOVAS with and without the inclusion of the confounders. As both HDI and years of education are considered proxies of cognitive reserve, we decided to use only the HDI variable because this variable reflects more accurately the quality of education in Brazil.

The MANCOVA analysis for the HCV study group (n = 28), after adjusting for the covariates (age, HDI, and sex), showed a statistically significant effect of HCV on the variables of the neuropsychological tests (F = 2.576, df = 6/36, *p* = 0.035, η^2^ = 0.300). The univariate tests showed that HCV affected RT (F = 4.184, df = 1/41, *p* = 0.047, η^2^ = 0.093) and VRT (F = 4.255, df = 1/41, *p* = 0.046, η^2^ = 0.094). In contrast, Commission Errors (F = 2.080, df = 1/41, *p* = 0.157, η^2^ = 0.048), Omission errors (F = 1.044, df = 1/41, *p* = 0.313, η^2^ = 0.025), SDMT (F = 0.268, df = 1/41, *p* = 0.608, η^2^ = 0.006), and COWAT (F = 0.258, df = 1/41, *p* = 0.614, η^2^ = 0.006), did not differ between the two groups (HCV vs. controls).

**Figure 2:**
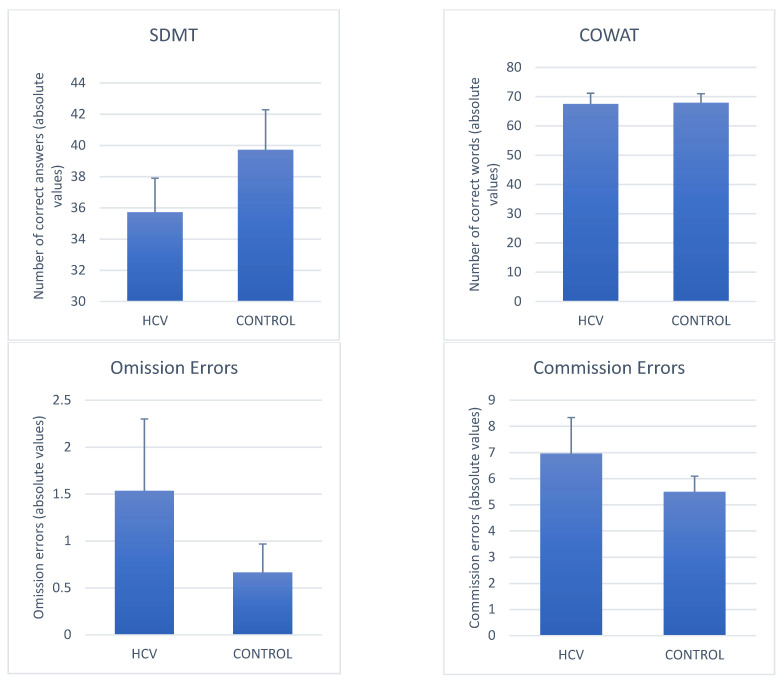

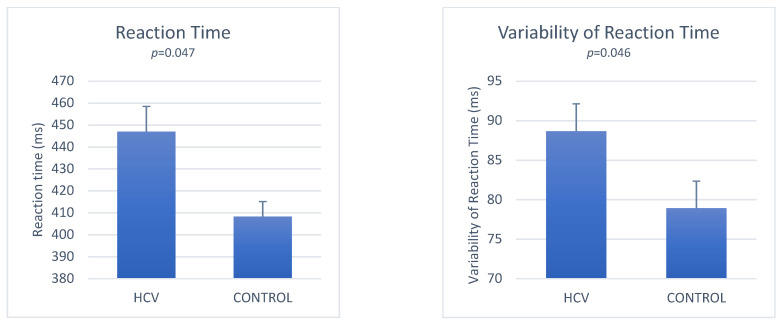
Comparison of the neurocognitive tests in HCV-Mild Hepatitis (n = 28) vs. Health Control group (n = 18). Values are adjusted means and each line represents the respective standard error of the mean. There are significant group differences only for the VRT and RT variables (*p* < 0.05). HCV, Hepatitis C Virus; SDMT, Symbol Digit Modalities Test; COWAT, Controlled Oral Word Association Test.

The results of the MANCOVA not adjusting for the confounders or covariates, showed the same results described for the MANCOVA with the three confounders. We found a statistically significant effect of HCV on the variables of the neuropsychological tests (F = 2.51, df = 6/39, *p* = 0.038, η^2^ = 0.28). The univariate tests also confirmed the results obtained using the three confounders.

#### 3.2.2. Variables That Discriminated Mild HCV from Controls

Considering all the neuropsychological variables (Omission errors, Commission Errors, RT, VRT, SDMT, Category, and Letters), we found one discriminating dimension. The function was found to have significant discriminant ability (71.7%). The smallest Wilk’s λ was found for RT. Accordingly, the pooled within-group correlations identified the largest correlations with the discriminant model for RT. The discriminant function correctly classified 71.7% of participants. The stepwise forward selection included only RT (Probability of F < 0.01).

### 3.3. Clinical Significance of the Differences

For the RT variable of the CVAT, 12 patients (42%) were at least 1SD worse than the controls. Regarding the normative means, 14 were above 1SD.

## 4. Discussion

HCV subjects without previous hepatic decompensation showed deficits in visuomotor reaction time (RT) and intraindividual variability of RT (VRT) as compared to healthy controls in a computerized attention task (CVAT). On average, no significant differences were observed in the other two variables of the CVAT (omission and commission errors) and in two traditional neuropsychological tests (COWAT and SDMT). A discriminant analysis indicated that the RT variable was the most suitable cognitive parameter to distinguish mild HCV patients from controls. Using stringent criteria to define minor cognitive impairment, which took into account both control and normative data, we found that 42% of the patients had a clinically significantly worse performance in the RT component of the visual attention system.

### 4.1. Verbal Fluency (COWAT)

Our results showed that there were no significant statistical differences in verbal fluency tests (COWAT) between HCV and controls. Karaivazoglou et al. [56], Huckans et al. [57], and Abrantes et al. [28] also did not find differences between HCV and control groups in verbal fluency. Thus, the present findings are in accordance with several previous studies and support the hypothesis that cognitive deficits in mild HCV subjects are restricted to certain cognitive domains.

### 4.2. SDMT

We found a non-significant worse performance in the HCV groups as compared to the control group. It should be mentioned that the mean of the control group (39.72 correct answers) is very similar to the normative data published in Latin America by Vanotti et al. [47]. These authors found a mean of 39.1 correct answers for subjects 51–70 years old. Our HCV initial sample (n = 48) and the subset with very mild liver disease (n = 28) obtained means of 32.35 and 35.71, respectively. The SDMT is a commonly used test to assess psychomotor speed. However, performance is also affected by visual scanning, tracking, motor speed, and working memory. Therefore, the multifactorial nature of the SDMT may have precluded the differences to reach statistical significance. Although we did not find statistically significant differences, our data suggest that the worse performance by the HCV groups may reflect a slower processing speed in the patients as compared to the healthy controls.

### 4.3. CVAT

In the neuropsychological evaluation based on the CVAT, we observed a worse performance of the four CVAT variables (omission error, commission error, RT, VRT) in the HCV group when compared to the control group. The univariate analysis indicated specific significant differences in RT and VRT. RT and VRT were both higher in HCV-infected subjects as compared to controls.

A compromised performance in the CVAT can be explained by four conditions: firstly, a drop in vigilance caused by falling adequate brain activation which causes slow Reaction Times (RT); secondly, occasional lapses in attention as the test progresses, affecting the stability of response times which causes an increase in the variability of the reaction times (VRT); thirdly, failure of focused attention, severe enough to result in omission errors; and fourthly, inability to control inadequate responses resulting in a high number of commission errors.

The number of omission errors and commission errors did not differ between HCV and controls. Therefore, putative attention deficits in the HCV group may be circumscribed to the attention subdomains associated with the speed of reaction, indicated by the significative differences in RT and VRT.

The higher VRT (sustained attention) exhibited by the HCV group might be explained by lapses in attention which affected the stability of response times and caused an increase in VRT [37,48]. Based on this assumption, our finding suggests that patients with chronic liver disease related to HCV exhibit sustained attention problems. However, VRT may also be related to RT and the sustained attention problems may reflect deficits in the intrinsic-alertness subdomain. The discriminant analysis gives support for the hypothesis that the results of VRT are explained by the RT variable. Therefore, we conclude that HCV subjects with mild liver disease exhibited sustained attention problems, secondary to a primary deficit associated with RT.

Previous functional neuroimaging studies using go/no-go tasks have indicated that RT is associated with the activation of the cingulate cortex and frontoparietal attention networks [34,35]. In addition, RT is linked to the brain stem arousal systems to keep alert. Furthermore, the neurotropism induced by the HCV infection is found in the frontal cortices, cingulate gyri, and medulla [11,32,33]. Taken together, these findings give support for the hypothesis that the observed RT impairment might be associated with the neurotropism of the HCV virus.

Our finding indicating a significant RT increase in HCV patients is also supported by Ibrahim et al. [58]. These authors found that the presence of HCV antibody-positive titer was a significant negative predictor factor for the reaction time evaluated in the Penn Continuous Performance Test, although the presence of HCV-RNA was not evaluated by polymerase chain reaction.

In addition, the interpretation of our intriguing finding on the RT parameter receives some support from previous neurophysiological studies. Abrantes et al. [30] found significative alterations of the P300 evoked potential in mild liver HCV-infected patients compared to controls. Ramchurn et al. [59] suggested that P300 alteration was associated with the speed of behavioral RTs and postulated that fluctuations in executive control underlie variability in speeding responses. Taken together we might suggest that the alterations of the P300 evoked potential described by Abrantes et al. [30] might reflect a deficit in the RT.

It is worth mentioning four good reasons to demonstrate that our results on RT are not restricted to mean differences. Firstly, the discriminant functions compared the whole distributions of the two groups (controls and HCV patients) and identified the RT variable as the most reliable discriminator. Secondly, using only the RT variable we were able to blindly predict an HCV patient with 71.7% accuracy. Thirdly, we found a significant percentage of patients with RT greater than 1 SD of the control means. Fourthly, a significant percentage of patients had RTs greater than 1 SD of the population mean.

### 4.4. Limitations and Strengths

In this study, the control group mostly consisted of females, and we could not exclude that some controls may have exhibited other comorbidities that might affect cognition. However, in the CVAT standardization study, females were found to be slower than males. Thus, our results reinforce our finding of a higher RT in HCV subjects.

Qualitative analysis indicates that the performance of the mild-HCV group (n = 28) was worse than the controls in all the neuropsychological variables except verbal fluency (COWAT). However, significance was reached only for the RT and VRT variables. The fact that we did not find significant differences in the other variables (OE, CE, and correct responses in the SDMT) may reflect the limitations associated with power and sample sizes. In the present investigation, the effect size for power analysis was specified to be the minimum meaningful effect, based on the clinical experience of the tests as suggested by Bakker et al., 2019 [60]. However, the commonly used interpretation is to refer to effect sizes as small (d = 0.2), medium (d = 0.5), and large (d = 0.8), where d is the standardized effect size. Therefore, we will need a larger sample size for the study of small or medium effects.

The absence of qualitative and quantitative differences in the COWAT suggests that further studies should be conducted testing HCV cases with a paired association word test rather than the COWAT. This should be particularly interesting as association tests have been shown to be sensitive for detecting occult cognitive deficits in subjects with preclinical Alzheimer’s disease [61,62]. The inclusion of other neuropsychological tests, such as the paired association word test, would allow an increase in the accuracy of the discriminant function.

One strength of our study was the strict inclusion criteria of the HCV group with most of the sample represented by subjects with mild liver disease (Fibrosis F0-F2), with normal liver synthesis tests, asymptomatic, and even with no subjective cognitive complaints at the interview. Thus, most of our HCV patients presented mild to moderate grade of fibrosis and no comorbidities. Another strength of this study was the interpretation of the clinical significance of the cognitive results considering both the normative means as well as the performance of the control group.

## 5. Conclusions

In mild liver disease (non-cirrhotic) HCV-infected subjects without a history of hepatic decompensation, there is a mild cognitive impairment circumscribed to the alertness subsystem. This impairment reaches a magnitude that has clinical significance.

## Data Availability

All the data is available and can be requested to the authors.
